# Assessing the Readability of Patient Education Materials on Cardiac Catheterization From Artificial Intelligence Chatbots: An Observational Cross-Sectional Study

**DOI:** 10.7759/cureus.63865

**Published:** 2024-07-04

**Authors:** Benjamin J Behers, Ian A Vargas, Brett M Behers, Manuel A Rosario, Caroline N Wojtas, Alexander C Deevers, Karen M Hamad

**Affiliations:** 1 Department of Internal Medicine, Sarasota Memorial Hospital, Sarasota, USA; 2 Department of Clinical Research, University of South Florida Morsani College of Medicine, Tampa, USA; 3 Department of Clinical Research, University of Florida, Gainesville, USA

**Keywords:** meta ai, google gemini, microsoft copilot, chatgpt, patient education materials, cardiac catheterization, readability, artificial intelligence

## Abstract

Background: Artificial intelligence (AI) is a burgeoning new field that has increased in popularity over the past couple of years, coinciding with the public release of large language model (LLM)-driven chatbots. These chatbots, such as ChatGPT, can be engaged directly in conversation, allowing users to ask them questions or issue other commands. Since LLMs are trained on large amounts of text data, they can also answer questions reliably and factually, an ability that has allowed them to serve as a source for medical inquiries. This study seeks to assess the readability of patient education materials on cardiac catheterization across four of the most common chatbots: ChatGPT, Microsoft Copilot, Google Gemini, and Meta AI.

Methodology: A set of 10 questions regarding cardiac catheterization was developed using website-based patient education materials on the topic. We then asked these questions in consecutive order to four of the most common chatbots: ChatGPT, Microsoft Copilot, Google Gemini, and Meta AI. The Flesch Reading Ease Score (FRES) was used to assess the readability score. Readability grade levels were assessed using six tools: Flesch-Kincaid Grade Level (FKGL), Gunning Fog Index (GFI), Coleman-Liau Index (CLI), Simple Measure of Gobbledygook (SMOG) Index, Automated Readability Index (ARI), and FORCAST Grade Level.

Results: The mean FRES across all four chatbots was 40.2, while overall mean grade levels for the four chatbots were 11.2, 13.7, 13.7, 13.3, 11.2, and 11.6 across the FKGL, GFI, CLI, SMOG, ARI, and FORCAST indices, respectively. Mean reading grade levels across the six tools were 14.8 for ChatGPT, 12.3 for Microsoft Copilot, 13.1 for Google Gemini, and 9.6 for Meta AI. Further, FRES values for the four chatbots were 31, 35.8, 36.4, and 57.7, respectively.

Conclusions: This study shows that AI chatbots are capable of providing answers to medical questions regarding cardiac catheterization. However, the responses across the four chatbots had overall mean reading grade levels at the 11^th^-13^th^-grade level, depending on the tool used. This means that the materials were at the high school and even college reading level, which far exceeds the recommended sixth-grade level for patient education materials. Further, there is significant variability in the readability levels provided by different chatbots as, across all six grade-level assessments, Meta AI had the lowest scores and ChatGPT generally had the highest.

## Introduction

Artificial intelligence (AI) is a burgeoning new field that has increased in popularity over the past couple of years, coinciding with the public release of large language model (LLM)-driven chatbots. Open AI’s ChatGPT has been the most widely used of these chatbots since its initial release in late 2022. Since then, numerous competing chatbots have been released by other companies, including Google Gemini, Microsoft Copilot, and Meta AI. LLM-driven chatbots can be engaged directly in conversation, allowing users to ask them questions or issue other commands. Since LLMs are trained on large amounts of text data, including encyclopedic resources, they can also answer questions reliably and factually [1]. This ability has allowed them to serve as a source for medical inquiries, with the possibility of replacing popular search engines such as Google [1].

Previously, the expansion of wireless internet and the proliferation of smartphones resulted in a substantial increase in the use of online, website-based patient education materials. Many of these websites are hosted by major institutions, such as government agencies, hospital systems, or universities. Assessments of the quality and readability of these websites have been performed on virtually every medical topic to date. Quality has generally been assessed using the DISCERN instrument, whereas various scores and grade-level assignments were used for readability [2]. Although the quality of resources is important, including their comprehensiveness and accuracy, it is also vital that these resources can be understood by the general public. Accordingly, the American Medical Association and National Institutes of Health have recommended that these materials not exceed a sixth-grade reading level [3,4].

Despite the abundance of studies examining website-based resources, few evaluations have been performed on the responses generated by AI chatbots in their default states. Given the increasing use of these chatbots, such evaluation is warranted to ensure they provide comprehensible health information to the general public. This study seeks to assess the readability of patient education materials on cardiac catheterization across four of the most common chatbots: ChatGPT, Microsoft Copilot, Google Gemini, and Meta AI.

## Materials and methods

We reviewed the top Google Search results for “cardiac catheterization” to familiarize ourselves with the layout of these website-based patient education materials, from which a set of 10 questions to ask the chatbots was developed (Table 1).

**Table 1:** The 10 standardized questions that we asked each chatbot.

Questions
What is cardiac catheterization?
Why is cardiac catheterization needed?
What happens during cardiac catheterization?
What are the benefits of cardiac catheterization?
What are the risks of cardiac catheterization?
How do I prepare for cardiac catheterization?
What happens before cardiac catheterization?
What happens after cardiac catheterization?
What type of results do you get from cardiac catheterization?
When should you call your doctor after cardiac catheterization?

We asked these 10 questions in consecutive order to each of the four chatbots: ChatGPT Version 3.5 (OpenAI, San Francisco, CA), Microsoft Copilot (Microsoft, Redmond, WA), Google Gemini 1.0 Pro (Google DeepMind, London, England, UK), and Meta AI Version 24.0 (Meta, New York, NY). The questions and subsequent answers were copied into a separate Microsoft Word (version 15.30) document for each of the chatbots. Each Word document then had all formatting and hyperlinks removed, as well as any citation numbers in the texts of the answers. This text was then copied into the Readable software (Readable.com, Horsham, UK) and analyzed across all of their readability models [5].

The Flesch Reading Ease Score (FRES) was used to assess readability. FRES provides a score between 0 and 100, with higher scores indicating better readability, and a target score of 60 or more [5-8]. Readability grade levels were assessed using six tools: Flesch-Kincaid Grade Level (FKGL), Gunning Fog Index (GFI), Coleman-Liau Index (CLI), Simple Measure of Gobbledygook (SMOG) Index, Automated Readability Index (ARI), and FORCAST Grade Level. All six tools provide a reading grade level corresponding to the number of years of US education necessary to understand the material [5-13]. These seven measures are widely used to analyze the readability of technical material. However, they all use different variables from the text of interest to assess its readability. A summary of each tool, including its formula, is provided in Table 2.

**Table 2:** A summary of the formats and formulas of the seven readability measures used in our study.

Readability Tool/Measure	Format/Purpose	Formula
Flesch Reading Ease Score (FRES) [5-8]	Assesses readability by providing a score between 1 and 100 Uses mean words per sentence and mean syllables per word Higher scores indicate better readability, with a target score of 60 or more Scoring of 70 to 80 is equivalent to an 8th-grade reading level, which means it should be fairly easy for the average adult to read	FRES = 206.835 – 1.015(TW/TS) – 84.6(TSy/TW) TW = Total Words TS = Total Sentences TSy = Total Syllables
Flesch-Kincaid Grade Level (FKGL) [5-8]	Assesses the approximate US reading grade level of a text on a scale from 0 to 18 Uses mean words per sentence and mean syllables per word Scores of 0 to 6 are considered basic reading level, while scores of 6 to 12 are average, and scores of 12 to 15 are advanced Material meant for the general public should be written at around an 8^th^ grade level	FKGL = 0.39(TW/TS) + 11.8(TSy/TW) – 15.59 TW = Total Words TS = Total Sentences TSy = Total Syllables
Gunning Fog Index (GFI) [5,6,9]	Estimates the education level needed to understand a text by providing a grade level between 0 and 20 Uses mean words per sentence and proportion of complex words (containing 3+ syllables) to total words A score of 6 is easily readable for sixth-graders, while scores over 17 are graduate-level Text for the general public should aim for a score of 8	GFI = 0.4 x [(TW/TS) + 100(CW/TW)] TW = Total Words TS = Total Sentences CW = Complex Words (3 or more syllables)
Coleman-Liau Index (CLI) [5,6,10]	Shows the approximate US reading grade level needed to understand a text Uses mean number of letters per 100 words and mean number of sentences per 100 words Focuses on long words and long sentences Requires texts to be at least 100 words long Content for the general public should aim for scores of 8 to 10	CLI = (0.0588 x L) – (0.296 x S) – 15.8 L = average number of letters per 100 words S = average number of sentences per 100 words
Simple Measure of Gobbledygook (SMOG) Index [5,6,11]	Measures the number of years of education an average person needs to understand a text Uses the number of words with 3+ syllables across 30 sentences from the text (10 from the beginning, 10 in the middle, and 10 at the end) Aim is for a score around 7-8	SMOG = 3 + √(Polysyllabic count) Polysyllabic count = number of words with 3+ syllables
Automated Readability Index (ARI) [5,6,12]	Assesses the US reading grade level of a text Uses mean number of characters per word and mean number of words per sentence Focuses on long words and long sentences Goal is to aim for a score of 9 or less, which corresponds to an 8th-grade reading level	ARI = 4.71(TC/TW) + 0.5(TW/TS) – 21.43 TC = Total Characters TW = Total Words TS = Total Sentences
FORCAST Grade Level [5,13]	Indicates the number of years of US education a reader needs to understand a text Uses the number of single-syllable words in a 150-word sample Can not calculate below a 5th-grade level Goal for materials meant for the general public is a grade of 9 or 10	FORCAST = 20 – (N/10) N = number of single-syllable words in a 150-word sample

The seven readability scores were input into a Microsoft Excel (version 15.30) document. All statistical analyses were carried out in this document. Mean and standard deviation were calculated for each of the six reading grade-level tools across all four chatbots. Mean and standard deviation for the overall grade level for each chatbot were also calculated. This was done by averaging the six reading grade-level scores for each chatbot.

## Results

Mean FRES across all four chatbots was 40.2, corresponding to a college reading level (FRES of 30-50) and thus being deemed difficult to read. Overall, the mean grade levels for the four chatbots were 11.2, 13.7, 13.7, 13.3, 11.2, and 11.6 across the Flesch-Kincaid, Gunning Fog, Coleman-Liau, SMOG, Automated Readability, and FORCAST indices, respectively. These results indicate that the material was written at the 11th- to 13th-grade level or that a reader will have needed to complete 11-13 years of US education to understand the content. Thus, overall, these patient education materials were written at a high school junior or college freshman reading level. The readability scores for each chatbot are provided in Table 3.

**Table 3:** Readability scores across all seven instruments. Mean (SD) for each score across all four chatbots is in row 6. The mean (SD) grade level across all six measures of reading grade level can be seen in column 9. FRES = Flesch Reading Ease Score; FKGL = Flesch-Kincaid Grade Level; GFI = Gunning Fog Index; CLI = Coleman-Liau Index; SMOG = Simple Measure of Gobbledygook Index; ARI = Automated Readability Index; FORCAST = FORCAST Grade Level; GL = Grade Level

Chatbot	FRES	FKGL	GFI	CLI	SMOG	ARI	FORCAST	Average GL
ChatGPT	31	14.1	17.3	15	15.9	14.8	11.8	14.8 (1.8)
Microsoft Copilot	35.8	10.9	12.6	14.8	12.5	10.6	12.2	12.3 (1.5)
Google Gemini	36.4	11.9	14.4	14.6	14.1	12	11.7	13.1 (1.4)
Meta AI	57.7	7.8	10.3	10.5	10.8	7.2	10.7	9.6 (1.6)
Average Across Chatbots	40.2 (11.9)	11.2 (2.6)	13.7 (3.0)	13.7 (2.2)	13.3 (2.2)	11.2 (3.2)	11.6 (0.6)	

ChatGPT had a FRES of 31, indicating that it was written at a college reading level. An average grade level across the six assessments was 14.8 with a standard deviation of 1.8, also indicating a college level. The lowest reading grade level for ChatGPT was 11.8 on FORCAST, while GFI was the highest at 17.3. These results suggest that at least 11.8 and as many as 17 years of US education would be required for the reader to comprehend the material, corresponding to high school junior and college graduate degree levels, respectively.

Microsoft Copilot had a FRES of 35.8 and an average reading grade level of 12.3, corresponding to being written at a college reading level. The standard deviation of this mean was 1.5. Microsoft Copilot’s lowest reading grade level was 10.6 on ARI, while the highest was 14.8 on CLI. These results suggest that anywhere from 10.6 to 14.8 years of US education would be required to understand the material, corresponding to high school sophomore and college sophomore levels, respectively.

Google Gemini had a FRES of 36.4, deeming this material to be written at a college level. An average grade level of 13.1 with a standard deviation of 1.4 across the six tools indicated that the material was written at a college sophomore level. The lowest reading grade level for Google Gemini was 11.7 on FORCAST and the highest was 14.6 using CLI. These results suggest that 11.7-14.6 years of US education are required to comprehend this material, corresponding to high school junior and college sophomore levels, respectively.

Meta AI had a FRES of 57.7, suggesting the material was written at the 10th-12th grade level. Across the six grade-level assessments, an average grade level of 9.6 with a standard deviation of 1.6 was obtained, representing a ninth-grade reading level. Meta AI had a low of 7.2 with ARI and a high of 10.8 using simple measure of Gobbledygook (SMOG). These results suggest that 7.2-10.8 years of US education are necessary to understand this material, corresponding to seventh grade and high school sophomore levels, respectively.

**Figure 1:**
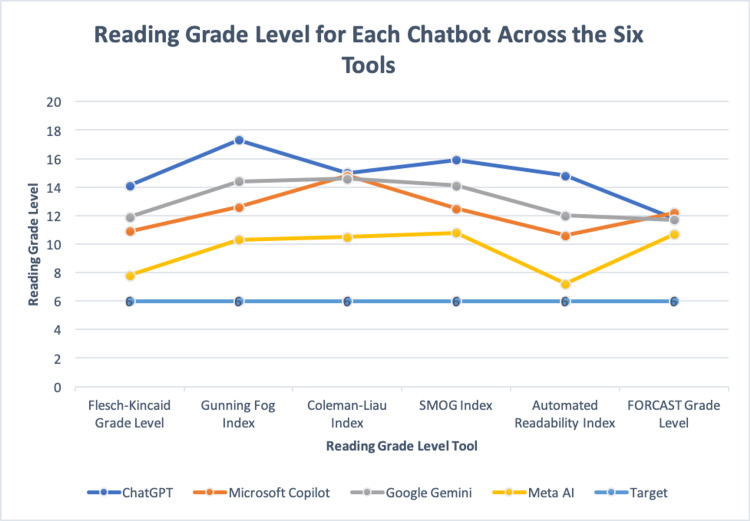
Graphical representation of the six reading grade level scores for each AI chatbot. A horizontal line was added to grade level six to show the target reading level.

In summary, Meta AI had the best readability scores across all seven measures, while ChatGPT generally had the worst. The only measure in which ChatGPT did not have the worst readability score was the FORCAST grade level, where Microsoft Copilot had a higher score. Better readability is represented by higher FRES values and lower reading grade level scores, as fewer years of US education are needed to understand the materials. The highest variability amongst grade level scores was seen with ChatGPT, while Google Gemini had the lowest. A graphical representation of the reading grade level across the measures for each chatbot can be seen in Figure 1.

## Discussion

Our results suggest that the information on cardiac catheterization provided by chatbots generally far exceeds the recommended sixth-grade reading level. Across the four chatbots, the mean FRES was 40.2, which is below the goal of 60 and suggests that the material is college-level reading. The average grade level was 11 or higher across all six measures, with the lowest being a mean Flesch-Kincaid of 11.2. Further, the averages across the GFI, CLI, and SMOG index all obtained grade levels over 13, again suggesting college-level reading material. However, the results were not consistent across all four chatbots. Meta AI had the best readability scores, while ChatGPT generally had the worst. Meta AI had a FRES of 57.7, nearly achieving the goal of over 60, and an average grade reading level of 9.6 across the six instruments. Its FKGL and ARI yielded grade levels of 7.8 and 7.2, respectively, which are closer to the goal of the sixth-grade level.

Moreover, the average readability provided by chatbots appears to be worse compared to materials found in website-based resources. A 2012 study of cardiac catheterization resources across 64 websites noted a mean FRES of 47.5, compared to our mean of 40.2 [14]. Furthermore, 12.5% of these websites were determined to have an FRES over the goal of 60, whereas none of the responses from our chatbots reached this threshold [14]. As with our findings, significant variability was observed between the websites with a range FRES of 9-66.7 [14].

Our readability findings are concerning given the increasing use of AI chatbots amongst the public. This concern has been corroborated by other studies that examined the readability of medical explanations provided by chatbots. One such study asked ChatGPT questions from the American Heart Association about atrial fibrillation, obtaining a mean reading grade level of 14.23 [15]. Another study asked ChatGPT questions about the use of platelet-rich plasma therapy for osteoarthritis and obtained a mean reading grade level of 17.18 using ChatGPT-3.5 and 16.36 with ChatGPT-4 [16]. These results suggest that chatbots may provide explanations at a higher-than-desired reading level across a range of medical questions.

It is also important to note that our chatbots were asked questions without providing identifying information that could impact the reading level. For example, the prior study on atrial fibrillation altered ChatGPT’s outputs by identifying as a patient and then as a physician, achieving a reduction in grade reading level to 12.81 and an increase to 16.73, respectively [15]. Although chatbots generally issue explanations that exceed the recommended grade level, at least in their default state, prior studies have shown that they can remedy this. Two studies showed ChatGPT and Google Bard/Gemini being able to effectively summarize and improve the readability of website-based patient education materials by copying their information and asking for translation to a desired reading level [17,18]. These findings are promising for the future of AI chatbots to provide patient education materials at the recommended reading level. However, studies have shown that ChatGPT’s ability to assess the readability of text differs significantly from established tools, such as Readable.com [19]. This may pose problems when using these instruments to simplify information into more readable forms.

There may also be considerable variability between chatbots in their propensity to simplify information. We saw this in our study with the variations across the four chatbots in the readability of information provided in response to the same questions. Similar findings were also demonstrated in a previously mentioned study as ChatGPT demonstrated significantly easier readability than Google Bard/Gemini despite both being prompted to translate the same information to a fifth-grade level [17]. Another source of variability is the existence of numerous models for each chatbot platform. In the abovementioned study on platelet-rich plasma therapy for osteoarthritis, different grade-level scores were obtained for answers generated by ChatGPT-3.5 (17.18) and ChatGPT-4 (16.36) [16]. Further, another study tasking ChatGPT-3.5 and ChatGPT-4 with creating patient education handouts at a specified grade level found instances where both versions outperformed each other [20]. Similarly, another study found that ChatGPT-4.0’s responses to frequently asked questions about hyperlipidemia were more readable than those from ChatGPT-3.5, while there was no difference between the two in terms of accuracy [21].

In addition to the concerns regarding the readability of responses, there is also the matter of the accuracy and comprehensiveness of the answers. One study on frequently asked questions about breast imaging found that Gemini and Microsoft Copilot were easier to read than ChatGPT, while ChatGPT had greater accuracy in its responses [22]. This suggests that caution must be used when obtaining medical knowledge from AI chatbots as the platforms are not uniformly accurate or readable. Further, these instruments are constantly being updated and changed, so the information provided is likely fluid and can only be stated for a single point in time.

This study is not without its limitations, the first of which is that AI chatbots are constantly evolving, so our results are only reflective of the point in time that we conducted this study and may not be reproducible. Further, given that we only performed each query once, there is a chance that readability could vary between generated responses that were not captured by our study. Additionally, we asked 10 specific questions in sequence, regardless of the previous answers, which likely differs from how the average person will utilize these tools and could have impacted the outputs of the chatbots. The use of only 10 questions for our assessment represents another limitation. It is also possible that the redundancy in the answers we solicited contributed to the lower readability assessments that we obtained. This possibility could have been mitigated had we done separate analyses for each answered question and combined these individual results for each chatbot. We are also unable to comment on anything about the responses to our questions outside of the readability, including their quality and comprehensiveness. Future studies should address this by formally assessing the content of the answers, as well as how they compare between different chatbots. Finally, we only interacted with these chatbots in their default states while asking questions without contextual prompting regarding our identities or desired outputs. There is also the matter of individualized models existing within platforms, wherein users can provide specific information regarding their identity and preferences that can tailor the chatbot’s outputs. Future studies could see whether modified prompts or individualized models could provide answers at an ideal reading level.

## Conclusions

This study shows the capability of AI chatbots to answer medical questions regarding cardiac catheterization. However, the responses across the four chatbots had overall mean reading grade levels at the 11th- to 13th-grade level, depending on the tool used. This means that the materials were at the high school and even college reading level, which far exceeds the recommended sixth-grade level for patient education materials. Additionally, there is significant variability in the readability levels provided by different chatbots, as Meta AI produced results with the best readability scores, while ChatGPT generally had the worst. These readability limitations may be mitigated through the ability of AI chatbots to alter the readability levels of the information they provide. Given that the AIs are constantly evolving, future studies should monitor their progress in providing accurate, comprehensible information to patients about cardiac catheterization. Creators of these instruments should seek to provide more comprehensible data at a lower reading grade level without compromising the quality of the information provided.

## References

[1] Golan R, Reddy R, Ramasamy R (2024). The rise of artificial intelligence-driven health communication. Transl Androl Urol.

[2] Reddy RV, Golan R, Loloi J, Diaz P, Saltzman RG, Watane A, Ramasamy R (2022). Assessing the quality and readability of online content on shock wave therapy for erectile dysfunction. Andrologia.

[3] Weiss BD (2003). Health Literacy: A Manual for Clinicians. http://lib.ncfh.org/pdfs/6617.pdf.

[4] Thomas ND, Mahler R, Rohde M, Segovia N, Shea KG (2023). Evaluating the readability and quality of online patient education materials for pediatric ACL tears. J Pediatr Orthop.

[5] (2024). Readability formulas. https://readable.com/readability/readability-formulas/.

[6] (2024). Readability tests. https://help.siteimprove.com/support/solutions/articles/80000448325-readability-tests.

[7] Flesch R (1948). A new readability yardstick. J Appl Psychol.

[8] Kincaid JP, Fishburne RP, Rogers RL, Chissom BS (1975). Derivation of New Readability Formulas (Automated Readability Index, Fog Count and Flesch Reading Ease Formula) For Navy Enlisted Personnel. https://stars.library.ucf.edu/istlibrary/56.

[9] Gunning R (1952). The Technique of Clear Writing. https://books.google.com/books/about/The_Technique_of_Clear_Writing.html?id=ofI0AAAAMAAJ.

[10] Coleman M, Liau TL (1975). A computer readability formula designed for machine scoring. J Appl Psychol.

[11] McLaughlin HG (1969). SMOG grading: a new readability formula. J Read.

[12] Caylor JS, Stitch TG, Fox LC (2024). Methodologies for determining reading requirements military occupational specialties. https://apps.dtic.mil/sti/citations/AD0758872.

[13] Vivian AS, Robertson EJ 2nd (1980). Readability of patient education materials. Clin Ther.

[14] Kirthi V, Modi BN (2012). Coronary angioplasty and the internet: what can patients searching online expect to find?. J Interv Cardiol.

[15] Lee TJ, Campbell DJ, Rao AK (2024). Evaluating ChatGPT responses on atrial fibrillation for patient education. Cureus.

[16] Fahy S, Niemann M, Böhm P, Winkler T, Oehme S (2024). Assessment of the quality and readability of information provided by ChatGPT in relation to the use of platelet-rich plasma therapy for osteoarthritis. J Pers Med.

[17] Rouhi AD, Ghanem YK, Yolchieva L (2024). Can artificial intelligence improve the readability of patient education materials on aortic stenosis? A pilot study. Cardiol Ther.

[18] Patel EA, Fleischer L, Filip P (2024). The use of artificial intelligence to improve readability of otolaryngology patient education materials. Otolaryngol Head Neck Surg.

[19] Golan R, Ripps SJ, Reddy R (2023). ChatGPT’s ability to assess quality and readability of online medical information: evidence from a cross-sectional study. Cureus.

[20] Lambert R, Choo ZY, Gradwohl K, Schroedl L, Ruiz De Luzuriaga A (2024). Assessing the application of large language models in generating dermatologic patient education materials according to reading level: qualitative study. JMIR Dermatol.

[21] Lee TJ, Rao AK, Campbell DJ, Radfar N, Dayal M, Khrais A (2024). Evaluating ChatGPT-3.5 and ChatGPT-4.0 responses on hyperlipidemia for patient education. Cureus.

[22] Tepe M, Emekli E (2024). Assessing the responses of large language models (ChatGPT-4, Gemini, and Microsoft Copilot) to frequently asked questions in breast imaging: a study on readability and accuracy. Cureus.

